# Effects of circulatory arrest and cardiopulmonary bypass on cerebral autoregulation in neonatal swine

**DOI:** 10.1038/s41390-021-01525-3

**Published:** 2021-05-04

**Authors:** Jonah A. Padawer-Curry, Lindsay E. Volk, Constantine D. Mavroudis, Tiffany S. Ko, Vincent C. Morano, David R. Busch, Tami M. Rosenthal, Richard W. Melchior, Brandon C. Shade, Kellie L. Schiavo, Timothy W. Boorady, Alexander L. Schmidt, Kristen N. Andersen, Jake S. Breimann, Jharna Jahnavi, Kobina G. Mensah-Brown, Arjun G. Yodh, Christopher E. Mascio, Todd J. Kilbaugh, Daniel J. Licht, Brian R. White, Wesley B. Baker

**Affiliations:** 1 Department of Pediatrics, Children’s Hospital of Philadelphia and the Perelman School of Medicine at the University of Pennsylvania, Philadelphia, PA USA; 2 Division of Cardiothoracic Surgery, Children’s Hospital of Philadelphia and the Perelman School of Medicine at the University of Pennsylvania, Philadelphia, PA USA; 3 Department of Physics and Astronomy, University of Pennsylvania, Philadelphia, PA USA; 4 Department of Anesthesiology and Pain Management, Department of Neurology, University of Texas Southwestern Medical Center, Dallas, TX USA; 5 Department of Perfusion Services, Children’s Hospital of Philadelphia, Philadelphia, PA USA; 6 Department of Anesthesiology and Critical Care Medicine, Children’s Hospital of Philadelphia and the Perelman School of Medicine at the University of Pennsylvania, Philadelphia, PA USA

## Abstract

**Background:**

Cerebral autoregulation mechanisms help maintain adequate cerebral blood flow (CBF) despite changes in cerebral perfusion pressure. Impairment of cerebral autoregulation, during and after cardiopulmonary bypass (CPB), may increase risk of neurologic injury in neonates undergoing surgery. In this study, alterations of cerebral autoregulation were assessed in a neonatal swine model probing four perfusion strategies.

**Methods:**

Neonatal swine (*n* = 25) were randomized to continuous deep hypothermic cardiopulmonary bypass (DH-CPB, *n* = 7), deep hypothermic circulatory arrest (DHCA, *n* = 7), selective cerebral perfusion (SCP, *n* = 7) at deep hypothermia, or normothermic cardiopulmonary bypass (control, *n* = 4). The correlation coefficient (LDx) between laser Doppler measurements of CBF and mean arterial blood pressure was computed at initiation and conclusion of CPB. Alterations in cerebral autoregulation were assessed by the change between initial and final LDx measurements.

**Results:**

Cerebral autoregulation became more impaired (LDx increased) in piglets that underwent DH-CPB (initial LDx: median 0.15, IQR [0.03, 0.26]; final: 0.45, [0.27, 0.74]; *p* = 0.02). LDx was not altered in those undergoing DHCA (*p* > 0.99) or SCP (*p* = 0.13). These differences were not explained by other risk factors.

**Conclusions:**

In a validated swine model of cardiac surgery, DH-CPB had a significant effect on cerebral autoregulation, whereas DHCA and SCP did not.

**Impact:**

- Approximately half of the patients who survive neonatal heart surgery with cardiopulmonary bypass (CPB) experience neurodevelopmental delays. This preclinical investigation takes steps to elucidate and isolate potential perioperative risk factors of neurologic injury, such as impairment of cerebral autoregulation, associated with cardiac surgical procedures involving CPB.
- We demonstrate a method to characterize cerebral autoregulation during CPB pump flow changes in a neonatal swine model of cardiac surgery.
- Cerebral autoregulation was not altered in piglets that underwent deep hypothermic circulatory arrest (DHCA) or selective cerebral perfusion (SCP), but it was altered in piglets that underwent deep hypothermic CBP.

## Introduction

Advances in neonatal cardiac surgical techniques and perioperative management have improved survival rates in children with complex congenital cardiac abnormalities.^1,2^ Despite this progress, however, abnormal neurodevelopment remains a common, long-term adverse outcome for these children.^3,4^ The prevalence of neurodevelopmental anomalies in neonates who underwent cardiac surgery with cardiopulmonary bypass (CPB) exceeds 30%,^3,5^ but the extent to which perioperative and intraoperative factors contribute to neurological injury is unknown, because mechanisms of neurologic injury during cardiac surgery are multifactorial and incompletely understood.^6,7^ One possible mediator of neurologic injury is impaired cerebral autoregulation, which is common after cardiac surgical procedures involving CPB.^8–10^ To study the effect of bypass strategy on autoregulation in a controlled environment, we investigated associations between CPB technique and intraoperative impairment of cerebral autoregulation in a neonatal swine model.

Cerebral autoregulation mechanisms help to maintain adequate and consistent cerebral blood flow (CBF) despite varying cerebral perfusion pressure.^11,12^ While the cerebral vasculature of neonates is physiologically and anatomically different from that of adults, the mechanisms that underlie autoregulation are still present, serving to maintain CBF at approximately 10–20 mL/100 g/min in healthy neonates.^12–16^ The physiologic stresses of surgery, deep hypothermia, and CPB can adversely affect these mechanisms,^17,18^ but the extent of these effects in newborns with complex congenital cardiac abnormalities is incompletely understood.^9,10^ Impairment of cerebral autoregulation may place patients at higher risk of hypo- or hyper-perfusion, with subsequent ischemia, cerebral edema, inflammatory injury, embolism, or hemorrhage. Cerebral autoregulation can be measured by correlating mean arterial blood pressure (MAP) with a metric of CBF.^19–21^ In subjects with impaired cerebral autoregulation, variations in MAP are strongly correlated with CBF, but when autoregulation mechanisms are intact, the influence of MAP on CBF is dampened.

Here, we used a neonatal swine model^22,23^ to reproduce the physiologic effects of neonatal CPB without the heterogeneity and multiple covariates of a clinical population. We compared the effects of four different surgical perfusion strategies on cerebral autoregulation: deep hypothermic circulatory arrest (DHCA), deep hypothermic cardiopulmonary bypass (DH-CPB), selective cerebral perfusion (SCP) at deep hypothermia, and normothermic cardiopulmonary bypass (control). Because prior methods to measure cerebral autoregulation suffer from poor signal to noise,^24^ partially due to the small magnitude of spontaneous MAP fluctuations,^20,25^ we developed methods to induce larger changes in MAP, and we only perform analysis on those time periods likely to have data with large signal-to-noise ratio. Our overall hypothesis was that DHCA would impair cerebral autoregulation more than other perfusion strategies.

## Methods

### Neonatal swine bypass model

All procedures were approved by the Institutional Animal Care and Use Committee at the Children’s Hospital of Philadelphia and were performed in concordance with the National Institutes of Health Guide for the Care and Use of Laboratory Animals. One-week-old piglets (*n* = 25) were used as the experimental model. This neonatal swine model offers comparable anatomic size and cortical maturation with the human neonate.^26^ An in-depth description of anesthetic, perioperative, operative, and perfusion management protocols has been published elsewhere.^22,23^ Briefly, the anesthetic protocol consisted of (a) a weight-based ketamine dose for induction; (b) inhaled isoflurane for maintaining anesthesia during intubation, neuromonitoring placement, and vascular access; (c) weight-based dosing of dexmedetomidine and fentanyl infusions for maintenance of anesthesia during CPB; and (d) a pH-stat strategy during cooling and hypothermia and an alpha-stat strategy during rewarming and postoperative care.

Cortical CBF was monitored using a laser Doppler probe (Periflux; Perimed, Ardmore, PA, USA) through a 2-mm burr hole placed 10 mm paramedian to the coronal suture and 10 mm posterior to the coronal suture and secured to the dura matter. MAP was measured via a pressure catheter (Millar Inc, Houston, TX, USA) in the femoral artery, and cortical intracranial temperature (ICT) was measured at approximately 5 mm below the cortical surface (Licox CC1-P1; Integra LifeSciences, Plainsboro, NJ, USA). Median sternotomy and atrio-aortic cannulation for CPB were performed, and CPB was commenced after achieving an activated coagulation time > 480 s via systemic heparinization. The target CPB flow rate was 150 mL/kg/min, and CPB management was adjusted as needed to maintain: (a) partial arterial oxygen pressures of >150 mmHg and carbon dioxide pressures in the range 35–45 mmHg, (b) pH within 7.35–7.45, (c) MAP > 35 mmHg, and (d) hematocrit > 28%.

**Fig. 1:**
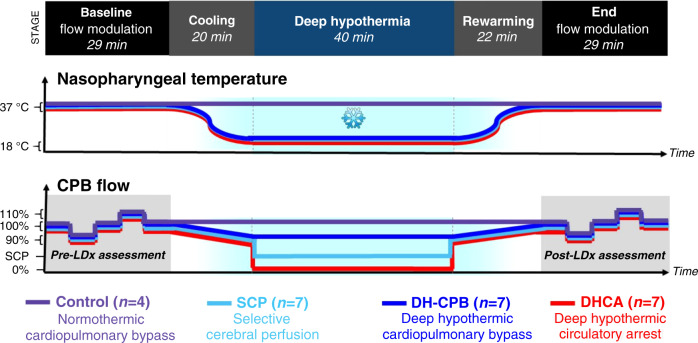
Schematic of nasopharyngeal temperatures and cardiopulmonary bypass (CPB) pump flow rates between the pre-intervention (Baseline) and post-intervention (End) CPB flow modulations used for cerebral autoregulation assessments. The temporal regions for autoregulation assessment are shaded in gray. Target flows for CPB were 150 mL/kg/min while selective cerebral perfusion (SCP) flows were targeted to 10 mL/kg/min. SCP was initiated after cooling and terminated before rewarming. During cooling, CPB flow rate was moderately lowered to between 125 and 150 mL/kg/min to maintain a MAP between 30 and 40 mmHg. Note, management prior to the first LDx measurement is not included in this schematic; it consisted of adjustments in anesthesia and vasoactive medications to maintain subject stability prior to measurements and the intervention. As shown in Table 1, this time prior to the experiment proper did vary between subjects by about 10 min.

Prior to anesthesia, piglets were randomized to DH-CPB (*n* = 7), DHCA (*n* = 7), SCP (*n* = 7), or control (*n* = 4) using block randomization (Fig. 1). It was not possible to blind researchers present during the experiment, but all data analysis was performed in a blinded fashion. DH-CPB, DHCA, and SCP subjects were cooled on full bypass flows to a nasopharyngeal temperature of 18 °C at a rate of no greater than 1 °C/min. Control animals were maintained at normothermia on CPB (normothermic CPB) for an identical duration as animals who underwent other perfusion strategies. For a period of 40 min, control subjects remained on full bypass flows, while DH-CPB subjects were maintained at a flow of 125–150 mL/kg/min to keep MAP between 30 and 40 mmHg. DHCA subjects underwent total circulatory arrest (i.e., zero bypass flow) for the same period. In the SCP cohort, following a brief period of circulatory arrest (<1 min), the aortic cannula was advanced into the right brachiocephalic artery and was circumferentially secured at the origin of the right carotid artery, at which point flows were resumed at 10 mL/kg/min to provide SCP for a period of 40 min. At the conclusion of this time, the aortic cannula was pulled back into the ascending aorta and flows were resumed at baseline levels. DH-CPB, DHCA, and SCP groups were all rewarmed on full bypass flows to normothermia at a rate no greater than 1 °C/min.

### Computation of pre- and post-intervention LDx cerebral autoregulation index

Cerebral autoregulation was assessed immediately prior to cooling and following rewarming (Fig. 1). To enhance signal to noise during each autoregulation assessment, bypass flow rates were modulated to induce changes in MAP (Fig. 1). Specifically, cerebral autoregulation was assessed using continuous CBF and MAP data; these data were acquired as the following bypass flow levels were achieved: 7 min  at baseline (150 mL/kg/min), 5 min at 90% of baseline (135 mL/kg/min), 7 min at baseline, 5 min at 110% of baseline (165 mL/kg/min), and 7 min at baseline (note, data acquired during the transitions between adjacent pump flow levels was also used; these transitions were about 20 s long).

**Fig. 2:**
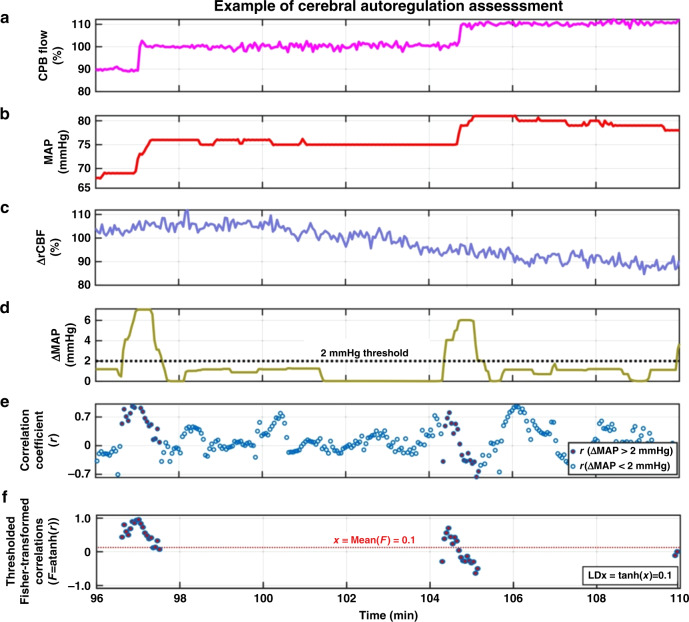
Case demonstration of how cerebral autoregulation was assessed in one piglet. The assessment used temporal measurements of MAP (**b**) and rCBF (**c**) during modulation of CPB flow (**a**). A 45-s moving window with maximal overlap was used to sample the data. For each window, the absolute change (maximum-minimum) in MAP (ΔMAP, **d**), and the Pearson correlation between MAP and rCBF (*r*, **e**) were calculated. The set of correlation coefficients for which ΔMAP exceeded 2 mmHg were then Fisher-transformed, and the hyperbolic tangent of their average returned a single LDx value for the assessment (**f**). In this piglet, rCBF was overall uncorrelated with MAP, resulting in an LDx of 0.1.

MAP and CBF were acquired during each cyclical pump flow manipulation (Fig. 2a–c); these data were smoothed with a 3-s Gaussian moving average filter and then resampled to 0.33 Hz. Since laser Doppler-measured CBF values are not calibrated, all CBF values were normalized to the average baseline level to generate relative CBF (rCBF). Next a continuous moving Pearson correlation coefficient was calculated between the MAP and rCBF data across maximally overlapping 45-s windows.

During periods of constant bypass flow rate, and thus relatively constant MAP, CBF should show only minimal physiologic variance. Consequently, variance from measurement noise should dominate the MAP and CBF data. As measurement noise is intrinsically uncorrelated, the averaged Pearson correlation coefficients (LDx) during such time intervals should have an expected value of zero regardless of whether autoregulation was intact or impaired. Therefore, we sought to develop a method that would quantify autoregulation via LDx only when physiologic variance in MAP (and possibly also CBF) was likely to be substantial. We therefore calculated the difference between the maximum and minimum values of MAP within each 45-s window, ΔMAP. We then applied a threshold such that only windows where ΔMAP exceeded 2 mmHg were included in further analysis (Fig. 2d, e). Furthermore, since the 2 mmHg ΔMAP threshold is somewhat arbitrary, we performed a sensitivity analysis using alternative ΔMAP thresholds (see ‘Statistical analysis’).

During each analysis period (i.e., pre- or post-intervention) the degree of cerebral autoregulation was quantified with an averaged LDx for that period. Since Pearson correlation coefficients are not normally distributed and are bounded by −1 and 1, the average was calculated after transformation of the correlation coefficients using Fisher transforms (*F* = arctanh(*r*), where *r* is the Pearson correlation coefficient, *F* is the Fisher-transformed value, and arctanh() is the hyperbolic arctangent) (Fig. 2f).^27,28^ The resulting averaged values were then transformed back to correlation space via the hyperbolic tangent (*r*_ave_ = tanh(*F*_ave_)) to yield a final averaged LDx for each subject during each measurement period.

Of note, LDx was originally developed^14^ as a moving correlation method to probe the same physiology as the pressure reactivity autoregulation index (PRx),^29^ which assesses the correlation between MAP and intracranial pressure. One assumption made in the use of LDx is that MAP is a proxy for cerebral perfusion pressure. Such an assumption is valid if intracranial pressure remains constant.

### Statistical analysis

Summary statistics are presented using means and standard deviations for normal data or medians and interquartile ranges for non-normal data. For all statistical tests, a *p* value of <0.05 was deemed to represent statistical significance.

#### Primary analysis: pre-intervention to post-intervention LDx comparisons across perfusion management strategies

Our main goal was to assess alterations in cerebral autoregulation among differing perfusion strategies. We assessed this using the difference between pre- and post-intervention LDx (ΔLDx). Specifically, an increase in LDx from pre- to post-intervention (i.e., ΔLDx > 0) was used to indicate that cerebral autoregulation is disrupted or more impaired. Our rationale was that an increase in LDx indicates that CBF changes are more closely related to MAP changes after the intervention, as compared to prior to intervention. This increased correlation would, in turn, suggest that autoregulation mechanisms are impaired (i.e., they are less able to compensate for MAP changes to sustain CBF). We assessed whether ΔLDx was significantly different from zero using Wilcoxon sign-rank tests. In a secondary analysis, Kruskal–Wallis tests were used to establish whether absolute pre-intervention and post-intervention LDx were significantly different across perfusion strategies. If significance was found, a post hoc Wilcoxon rank-sum test between each perfusion strategy was used.

#### Effect of other variables on LDx variation

We also set out to investigate whether other clinical variables could explain the observed variance in LDx. This analysis took two forms. First, we asked whether any pre-intervention variable could explain the observed variance in pre-intervention LDx values. Second, we asked whether any other factors associated with the intervention could help explain the post-intervention LDx values.

For the analysis of pre-intervention factors, we considered all piglets’ LDx values as a single cohort, because, at this point, there had been no intentional differences in management. Possible risk factors included in this analysis included anesthesia exposure (isoflurane, ketamine, dexmedetomidine, and fentanyl doses), the total time on bypass prior to the first LDx assessment, and the average MAP level during the assessments. Isoflurane cumulative dose was calculated by convolving the time spent at a certain percentage and the associated percentage (e.g., 2% at 10 min and 3% at 10 min would result in a cumulative dose of 20% min + 30% min for a total of 50% min). Analogous calculations were done for ketamine (units of mg/kg), dexmedetomidine (μg/kg), and fentanyl (μg/kg) infusions. All four anesthetics were regressed separately against pre-intervention LDx. We linearly regressed these possible explanatory variables against pre-intervention LDx and used *t*-statistics to test for significance.

We then assessed whether (in addition to changes in autoregulation) perfusion strategy altered other possible risk factors for poor postoperative outcomes. Specifically, we measured the absolute post-intervention values of MAP, hematocrit, pCO_2_, pO_2_, blood pH, lactate, and ICT. Since changes in ICT and in arterial pCO_2_ can influence autoregulation,^30,31^ we also examined their post-intervention minus pre-intervention changes (i.e., ΔICT and ΔpCO_2_). For each variable, an ANOVA was used to test for significant differences between perfusion strategies, and if significance was found for the ANOVA, a post hoc *t*-test between each cohort was used. As hypo- or hyper-perfusion may result in vascular and neuronal injury, intra-intervention CBF relative to baseline was calculated and compared between cohorts. A Kruskal–Wallace test was used to determine significant differences in intra-intervention CBF; if significance was found a post hoc Wilcoxon rank-sum test between each cohort was used.

#### Sensitivity of LDx results to window length and ΔMAP threshold

We based our choice of a 45-s window and a 2 mmHg ∆MAP threshold on the time course of expected physiologic variation and what was predicted to be a clinically relevant change. We recognize that these choices are somewhat arbitrary. We thus performed a sensitivity analysis of the effect on our LDx results of these particular choices. Accordingly, we reanalyzed the data, as described above, to determine LDx using different ΔMAP thresholds: either 0 mmHg (i.e., the prior standard of no threshold) or a threshold of 1, 2, or 3 mmHg (of note, ΔMAP variations >3 mmHg occurred too rarely for thresholds larger than 3 mmHg to be used). Similar re-analyses were performed for different window durations testing both windows of similar length to the induced MAP changes (i.e., 30, 45, and 60 s) as well as a longer window length (240 s as used in prior literature).^29^ We then assessed whether the use of different thresholds changed our conclusions about the LDx comparisons. Additionally, we performed an analysis of the differences between our method (45-s windows and a 2 mmHg threshold) and the prior standard (no threshold). These two sets of data were compared using a Kruskal–Wallace rank-sum test and a Bland–Altman analysis. For the Bland–Altman analysis, the mean difference and limits of agreement were computed in Fisher space, and then transformed back to correlation space.

We also asked whether our use of CPB pump flow manipulations was successful in generating substantial changes in ΔMAP. To this end, we assessed every pump flow change (i.e., 200 pump flow changes in total; 8 per animal as shown in Fig. 1), and we determined whether there was a 45-s window with a ΔMAP variation >2 mmHg that directly overlapped with the time of changing pump flow. The percentage of pump flow changes associated with windows with ΔMAP variation > 2 mmHg was computed. We further computed the percentage of all windows with ΔMAP variation >2 mmHg that overlapped with the pump flow change times.

## Results

Twenty-five piglets were included in the study, with seven randomized to each experimental condition (DH-CPB, SCP, and DHCA) and four randomized to the control group (normothermic bypass). The experimental groups did not differ in any clinical variables (Table 1), except for (a) SCP had a longer time on bypass prior to the initial LDx assessment than DH-CPB (44 ± 15 min compared to 25 ± 8 min; *p* = 0.01); (b) SCP had a lower cumulative dexmedetomidine dose than DHCA (2.7 ± 0.6 μg/kg compared to 4.8 ± 1.9 μg/kg; *p* = 0.02); and (c) DH-CPB had a lower hematocrit than control (31 ± 2% compared to 37 ± 2%; *p* < 0.01). In particular, pre-intervention LDx was similar between the groups (*p* = 0.33, Table 1).

**Table 1:** Pre-intervention experimental and clinical variables, presented as means and standard deviations or medians and interquartile ranges (*p* values were obtained from ANOVA or Kruskal–Wallace tests).

Variable	All subjects (*n* = 25)	DH-CPB (*n* = 7)	SCP (*n* = 7)	DHCA (*n* = 7)	Control (*n* = 4)	*p* value
LDx	0.27 [0.09, 0.56]	0.15 [0.03, 0.26]	0.56 [0.03, 0.26]	0.41 [0.10, 0.57]	0.34 [0.14, 0.61]	0.33
Weight (kg)	3.9 ± 0.5	4.2 ± 0.4	4.0 ± 0.2	3.6 ± 0.7	3.7 ± 0.2	0.06
Time on bypass prior to initial LDx assessment (min)	28.0 ± 8.0	25.0 ± 8.0	43.9 ± 15.2	31.9 ± 8.8	25.0 ± 4.8	0.01
Fentanyl (cumulative dose, μg/kg)	270 ± 95	249 ± 133	245 ± 35	323 ± 102	262 ± 64	0.41
Dexmedetomidine (cumulative dose, μg/kg)	3.5 ± 1.5	3.4 ± 1.4	2.7 ± 0.6	4.8 ± 1.9	2.7 ± 1.0	0.04
Isoflurane (cumulative dose, % × min)	351 ± 90	333 ± 57	323 ± 113	397 ± 91	351 ± 62	0.40
Ketamine (cumulative dose, mg/kg)	23.5 ± 4.9	22.9 ± 7.9	25.2 ± 1.4	23.5 ± 4.9	21.8 ± 0.9	0.73
Average MAP during pre-intervention LDx assessment (mmHg)	59.0 ± 7.9	57.5 ± 8.1	54.9 ± 4.2	64.0 ± 10.3	59.8 ± 4.1	0.17
Baseline nasopharyngeal temperature (°C)	37.2 ± 0.5	37.5 ± 0.4	36.8 ± 0.2	37.3 ± 0.7	37.4 ± 0.5	0.09
Baseline intracranial temperature (°C)	36 ± 2	34.6 ± 2.3	36.2 ± 0.6	36.3 ± 1.0	36.4 ± 1.4	0.12
pH	7.45 ± 0.06	7.45 ± 0.05	7.51 ± 0.04	7.42 ± 0.07	7.44 ± 0.06	0.06
Arterial pO_2_ (mmHg)	259 [204, 304]	211 [197, 320]	259 [232, 292]	235 [130, 94]	287 [203, 329]	0.80
Arterial pCO_2_ (mmHg)	42.4 ± 6.9	41.4 ± 3.8	39.1 ± 4.6	44.7 ± 10.9	45.8 ± 3.9	0.34
Arterial lactate (mM)	3.2 ± 0.9	3.2 ± 0.8	3.2 ± 0.5	3.3 ± 1.3	3.1 ± 0.7	0.99
Hematocrit (%)	33.6 ± 3.3	30.9 ± 2.0	33.9 ± 3.2	34.0 ± 3.2	37 ± 2.2	0.01

Using post hoc tests, SCP has a longer pre-intervention time on bypass than DH-CPB (*p*  =  0.01); SCP has a lower cumulative dexmedetomidine dose than DHCA (*p*  =  0.02); DH-CPB has a lower hematocrit than control (*p* <  0.01).

### Primary analysis: effects of perfusion strategy on LDx

**Fig. 3:**
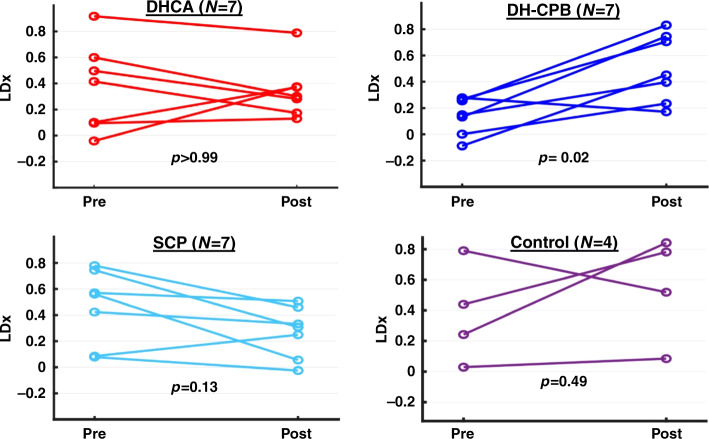
Pre- and post-intervention cerebral autoregulation for all piglets as evaluated by LDx. ΔLDx was significantly greater than zero only in the DH-CPB group.

Our main goal was to assess whether any perfusion strategy was associated with impairment in cerebral autoregulation (i.e., increases in LDx across the intervention: ΔLDx, defined as the post-intervention LDx minus the pre-intervention LDx). LDx significantly increased only after DH-CPB (*p* = 0.02, Table 2 and Fig. 3). All other groups did not demonstrate significant changes from pre- to post-intervention (Table 2 and Fig. 3). Of note, the absolute post-intervention LDx did not significantly differ between experimental groups (Table 2).

**Table 2:** Post-intervention measures of cerebral autoregulation for each perfusion strategy (presented as medians and interquartile ranges).

Variable	DH-CPB (*n* = 7)	SCP (*n* = 7)	DHCA (*n* = 7)	Control (*n* = 4)	*p* value
LDx	0.45 [0.27, 0.74]	0.31 [0.10, 0.43]	0.30 [0.20, 0.37]	0.65 [0.30, 0.81]	0.29
ΔLDx	0.52 [0.24, 0.65]	−0.11 [−0.52, −0.09]	−0.25 [−0.34, 0.22]	0.29 [−0.20, 0.64]	DHCA: >0.99 DH-CPB: 0.02 SCP: 0.13 Control: 0.38

ΔLDx is significantly greater than zero for DH-CPB group (*p* = 0.02).

### Analysis of other possible clinical risk factors

There was considerable variance in pre-intervention LDx. To determine whether this variance could be explained by pre-intervention clinical variables, we analyzed all piglets as a single cohort and assessed whether pre-intervention LDx was explained by any clinical variable using linear regression. No pre-interventional parameters were significantly associated with pre-intervention LDx (Table 3).

**Table 3:** Linear regression analyses of pre-interventional parameters versus pre-interentional LDx.

Pre-interventional parameter	Slope	*p* value
Time from start of bypass to baseline LDx measurement (per min)	0.005	0.253
Cumulative fentanyl dose (per μg/kg)	0.001	0.332
Cumulative dexmedetomidine dose (per μg/kg)	−0.030	0.438
Cumulative isoflurane dose (per %min)	0.001	0.854
Cumulative ketamine dose (per mg/kg)	0.010	0.434
Average mean arterial pressure (per mmHg)	−0.007	0.370

Statistical tests for a slope different from zero were done using a *t*-statistic.

We next assessed whether any clinical variables associated with the interventions differed between groups to possibly explain why cerebral autoregulation might be differentially affected by the perfusion strategies. As expected, piglets undergoing DHCA had near-zero relative CBF (rCBF) during the intervention (Table 4). The use of SCP also resulted in a significant decrease in rCBF (to a median of 35%), which was significantly lower (*p* = 0.03) than rCBF with DH-CPB (median 63%). No between-group differences were found for post-intervention MAP, pCO_2_, pO_2_, blood pH, lactate, ICT, ∆ICT, ΔpCO_2_, or hematocrit (Table 4).

**Table 4:** Intra- and post-intervention clinical variables (presented as means and standard deviations or medians and interquartile ranges) for the different perfusion strategies.

Variable	DH-CPB (*n* = 7)	SCP (*n* = 7)	DHCA (*n* = 7)	Control (*n* = 4)	*p* value
Intra-intervention relative cerebral blood Flow (%)	63 [40, 70]	35 [17, 38]	5 [4, 13]	75 [62, 93]	0.02
Changes in intracranial temperature (°C)	−1.3 ± 1.1	−1.7 ± 2.3	−0.7 ± 3.0	N/A	0.31
Changes in arterial pCO_2_ (mmHg)	4.3 ± 18.0	2.7 ± 10.1	−5.1 ± 8.1	−6.2 ± 6.7	0.82
Post-intervention mean arterial pressure (mmHg)	63.7 ± 22.9	56.0 ± 6.6	75.4 ± 9.9	58.1 ± 6.4	0.08
Post-intervention pH	7.4 ± 0.1	7.4 ± 0.1	7.4 ± 0.1	7.4 ± 0.0	0.70
Post-intervention Arterial pO_2_ (mmHg)	280 [140, 330]	230 [130, 250]	230 [170, 300]	280 [260, 320]	0.52
Post-intervention arterial pCO_2_ (mmHg)	45.7 ± 17.9	41.9 ± 7.7	39.6 ± 6.9	39.7 ± 3.4	0.73
Post-intervention arterial lactate (mM)	5.1 ± 1.7	6.7 ± 1.2	6.9 ± 1.8	6.0 ± 1.2	0.13
Post-intervention hematocrit (%)	35.3 ± 3.9	37.0 ± 4.3	33.7 ± 5.0	30.8 ± 4.4	0.17
Post-intervention intracranial temperature (°C)	33.3 ± 2.7	34.4 ± 2.2	35.5 ± 2.5	36.3 ± 1.7	0.20

Relative cerebral blood flow during the intervention was significantly different between groups.

### Sensitivity analysis

As previously mentioned, in order to improve signal to noise, we developed methods to calculate LDx only for time periods where MAP changed more than 2 mmHg. We compared these methods to those that would be found with prior standard methods (i.e., no ∆MAP threshold). Recall that the use of the threshold was to prevent LDx values being biased towards zero by periods where neither MAP nor CBF experienced substantial changes. We compared LDx for each piglet and each time point (pre- or post-intervention) calculated with the two methods. As expected, the median (IQR) LDx calculated without a threshold (0.17 [0.07, 0.30]) was significantly lower than the median LDx calculated with the threshold (0.32 [0.13, 0.56]; *p* < 0.01). The mean and 95% CI of their difference (i.e., LDx computed with threshold minus LDx computed without a threshold) was 0.24 (0.17, 0.32). Bland–Altman analysis also showed this bias towards zero arising from calculation of LDx without a MAP threshold (data not shown).

We then assessed whether our primary results were dependent on the particular MAP threshold or window length chosen. Neither the use of different window lengths (30, 45, 60, or 240 s) nor different ΔMAP thresholds (>1, >2, or >3 mmHg) for the computation of LDx changed the primary conclusions about LDx after the interventions (e.g., LDx significantly increased only after DH-CPB).

Finally, we found that our CPB pump flow manipulations were successful in generating greater changes in MAP. Every pump flow change (*N* = 200) overlapped with a 45-s window with ΔMAP > 2 mmHg. Furthermore, 60% of all windows with ΔMAP > 2 mmHg overlapped with the pump flow change times, while the remaining 40% of MAP changes > 2 mmHg were due to spontaneous MAP changes during periods of stable CPB flow rates.

## Discussion

While the choice of perfusion strategy during neonatal cardiac surgery would appear to be a modifiable risk factor for poor neurodevelopmental outcomes, prior clinical studies have failed to establish a benefit to choosing either DHCA or SCP.^6,32^ Clinical comparisons are hampered by patient heterogeneity as well as operative variation, which may be driven by surgeons’ preferences and accumulated experience. Our group has used a preclinical swine model of neonatal bypass surgery to reduce the heterogeneity seen clinically and to better isolate individual risk factors.^22,23^ In this contribution, we used this swine model to investigate the influence of bypass technique on cerebral autoregulation, which may be an important mediator of neurologic injury in critically ill infants.^8–10^ Our evidence suggests that DH-CPB has a greater propensity to impair cerebral autoregulation compared to other perfusion strategies.

Based on prior work, we expected the opposite: that DHCA would result in more impaired cerebral autoregulation compared to methods that preserve CBF. DHCA, for instance, has been associated with lactic acidosis and mitochondrial dysfunction,^22,33^ as well as with increased markers of tissue inflammation.^34^ Previous studies in piglets also showed that DHCA can impair physiologic vascular responses to hypercapnia and hypoxia.^35,36^ These factors can all potentially disrupt autoregulation. Our results, however, indicated that LDx significantly worsened following only deep hypothermic CPB and not DHCA.

One possible explanation for this result is a damaging effect of hyper-perfusion. CBF was substantially higher in the DH-CPB group than in the SCP group; while DH-CPB, as such, is not a valid strategy for children undergoing arch reconstruction, it can be taken as a proxy for *higher flow* SCP. The association between higher rCBF and impaired autoregulation supports the hypothesis that hyper-perfusion is harmful to the neonatal brain during bypass, a hypothesis also supported by prior piglet studies that found high flow SCP resulted in increased cerebral edema and injury.^37,38^ In our study, the bypass flow rate for SCP is on the lower end of the spectrum of flow rates typically used clinically, and practice patterns vary considerably.^34^ Furthermore, CPB or SCP flow rates are determined based on parameters such as MAP and inflow/outflow pressures in the bypass machine; these global physiologic measures do not necessarily represent the optimal flow rate to match cerebral metabolism. In the future, continuous neurologic monitoring using methods such as those presented here, or using diffuse optical methods,^14,21,23,39–43^ offers the potential to optimize bypass pump flow rates based on individualized physiologic monitoring.^44,45^

Another explanation for our results is that we, in contrast to some prior studies,^35,46,47^ measured post-intervention autoregulation immediately after rewarming rather than later in recovery. The negative physiologic effects of DHCA may be delayed and may not yet be apparent during the period assessed in this study. Additionally, evidence from previous work suggests that cerebral autoregulation is influenced by ICT.^18,48–51^ It is possible that the ability to maintain intracranial hypothermia with each technique is an important contributing factor; note, however, we did not observe differences in ICT between treatment groups. Thus, our results point to an under-recognized risk from DH-CPB, and they demonstrate the value for continuous non-invasive neuromonitoring during surgery, as well as the importance of high-fidelity models for translation.

We also observed considerable variability in pre-intervention LDx measurements across animals. We did not find any pre-operative variable that explained this variance; notably length of bypass prior to the measurement and anesthetic exposure did not affect LDx. These results demonstrate that even in a cohort of healthy piglets (i.e., without congenital heart disease), pre-operative physiology can vary, as is the case with human patients. Clinical studies have found variability in cerebral autoregulation for neonates in intensive care even in the absence of surgery.^52,53^ This high variability across subjects again highlights the need for real-time monitoring of cerebral autoregulation throughout the hospitalization of critically ill neonates and during the peri- operative period.

In addition to studying the effects of different bypass strategies, we also attempted to improve methodologies for calculating LDx. In many situations, MAP may vary only slightly; in such cases, variance due to measurement noise can be more significant than variance due to true physiology. Methods for calculating autoregulation that do not account for this problem will be biased towards a correlation coefficient of zero. That is, cerebral autoregulation may be falsely labeled as “intact” even if the true correlation coefficient in the presence of physiologic MAP variations would have been higher.^24^ We developed two methods to improve on prior standards and to better calculate a quantitative measure of autoregulation. First, to enhance the signal-to-noise ratio of our measurements of cerebral autoregulation, we only calculated LDx when changes in MAP were higher than a threshold. The use of a threshold resulted in increased calculated LDx values (representing the decreased influence of measurement noise). Secondly, during both the pre- and post-intervention LDx measurements, we imposed variations in the bypass flow rate to induce changes in MAP. While the overall effect on MAP of the pump flow manipulations was small, we did find that these changes resulted in an increase in the number of times when ΔMAP exceeded the threshold. Both methods are generalizable to other clinical and preclinical models, as well as other techniques for measuring CBF.

Herein, we have assessed changes in autoregulation (ΔLDx). We note, however, that other investigators have defined autoregulation in a binary fashion as “intact” or “impaired”.^10,14,54^ This binary approach relies on Lassen’s model of autoregulation,^55^ wherein blood flow is kept constant within a wide range of MAP levels between the lower and upper blood pressure limits of autoregulation. If MAP is within this range, autoregulation is intact, and if MAP is outside this range, autoregulation is impaired. However, as summarized in recent reviews,^11,30^ a growing volume of studies have found substantially more complex relationships between blood pressure and blood flow than that predicted by Lassen’s model. From this alternative point of view, the mechanisms of autoregulation function across multiple intermediate levels that range more continuously between fully intact and completely impaired.^21,56,57^ We adopted this alternative viewpoint when making our assessments of autoregulation after interventions. Since the level of variance in LDx across animals even prior to intervention was large, we felt that the binary classification was too simplistic. Thus, we do not make conclusions about autoregulation being fully “intact” prior to the intervention, and completely “impaired” after the intervention. Instead, we interpreted a significant increase in LDx from pre- to post-intervention as an impairment in cerebral autoregulation. It is important to note that neither the binary nor the continuous interpretation of autoregulation should be taken as definitive. Longer studies that follow neurodevelopmental outcomes will be required to determine whether there is increasing risk of poor outcomes from increasingly poor autoregulation and whether there is a threshold of correlation above which autoregulation should be considered impaired.

Our study had several limitations. First, our sample size was small, and we may be underpowered to detect differences that may exist between the surgical groups. In particular, increased impairment of autoregulation after DHCA might become apparent in a larger cohort. Additionally, variance in LDx from anesthetic exposure might be demonstrated with more subjects. We note that even the control group had a large amount of variance in LDx in this study. A larger study is warranted to more fully explain the sources of variance in LDx measurements. Note, others have shown that autoregulation can be influenced by arterial blood pCO_2_, ICT, absolute MAP level, or neuronal metabolism.^30,31^ While we did not find that any of these factors individually explained pre-intervention LDx, the large observed variance might be explained by a complex interplay between such factors. Note also, no significant differences were found between groups in arterial pCO_2_ changes, ICT changes, and post-intervention MAP levels; thus, we do not expect our conclusions to be impacted by such underlying physiological variance.

One assumption in the calculation of LDx is that MAP can serve as a proxy for cerebral perfusion pressure; this assumption is valid if intracranial pressure remains constant. If intracranial pressure was differentially affected by perfusion strategies, then our results could have been affected. Overall, these limitations demonstrate that absolute LDx may be difficult to interpret; this observation motivated our use of normalized individual changes, ΔLDx.

Future work is necessary to determine what metric for measuring autoregulation (e.g., ΔLDx, absolute LDx, limits of autoregulation, or dynamic autoregulation) is most appropriate for the neonatal population. Such a study should include an assessment of long-term neurodevelopmental outcomes or at least other biomarkers of injury that have been shown to be a proxy for such outcomes. Our small pilot study was inadequately powered to assess multiple definitions of autoregulation or to understand whether there are thresholds in absolute LDx that portend poor outcomes. Specifically, we note that the induced changes in MAP in this pilot study occurred too slowly to assess dynamic autoregulation effects in response to a step change in MAP.^56,58^

The present study focused solely on the effects of the intra-intervention period, but there is mounting evidence that the loss of cerebral autoregulation continues to have an impact in the immediate post-intervention period and beyond. A longer-term study is required to fully elucidate these critical relationships. Our results also capture only one possible risk factor for neurologic injury, and the negative effects of any given bypass strategy are not necessarily mediated through impairment of autoregulation.

In conclusion, using a neonatal swine model of different cerebral perfusion strategies, we found the unexpected result that immediate post-intervention cerebral autoregulation was more impaired by deep hypothermic circulatory bypass compared to DHCA. Additionally, improvements to the LDx method demonstrated here should allow more accurate measurement of autoregulation in both preclinical models and translational studies in human patients. Our future research will focus on elucidating the relationship between perfusion strategy, cerebral autoregulation, and other clinical variables.
